# The Transcriptional Cofactor MCAF1/ATF7IP Is Involved in Histone Gene Expression and Cellular Senescence

**DOI:** 10.1371/journal.pone.0068478

**Published:** 2013-07-30

**Authors:** Nobuhiro Sasai, Noriko Saitoh, Hisato Saitoh, Mitsuyoshi Nakao

**Affiliations:** 1 Department of Medical Cell Biology, Institute of Molecular Embryology and Genetics, Kumamoto University, Kumamoto, Japan; 2 Department of Biological Sciences, Graduate School of Science and Technology, Kumamoto University, Kumamoto, Japan; 3 Core Research for Evolutional Science and Technology (CREST), Japan Science and Technology Agency, Tokyo, Japan; National Cancer Institute (INCA), Brazil

## Abstract

Cellular senescence is post-mitotic or oncogene-induced events combined with nuclear remodeling. MCAF1 (also known as hAM or ATF7IP), a transcriptional cofactor that is overexpressed in various cancers, functions in gene activation or repression, depending on interacting partners. In this study, we found that MCAF1 localizes to PML nuclear bodies in human fibroblasts and non-cancerous cells. Interestingly, depletion of MCAF1 in fibroblasts induced premature senescence that was characterized by cell cycle arrest, SA-β-gal activity, and senescence-associated heterochromatic foci (SAHF) formation. Under this condition, core histones and the linker histone H1 significantly decreased at both mRNA and protein levels, resulting in reduced nucleosome formation. Consistently, in activated Ras-induced senescent fibroblasts, the accumulation of MCAF1 in PML bodies was enhanced via the binding of this protein to SUMO molecules, suggesting that sequestration of MCAF1 to PML bodies promotes cellular senescence. Collectively, these results reveal that MCAF1 is an essential regulator of cellular senescence.

## Introduction

Cellular senescence is a permanent cell cycle arrest that is induced by various stresses such as activated oncogenes, short telomeres, oxidative stress, and inadequate growth conditions [1]. In vivo evidence revealed that cellular senescence occurs in benign or premalignant lesions and acts as an important anti-tumor mechanism [2,3]. Senescent cells are characterized by several features including permanent cell cycle arrest, senescence-associated β-galactosidase (SA-β-gal) activity, morphological changes, activation of DNA damage signaling, and expression of cytokines or secreted factors [1]. Dynamic chromatin changes, including the formation of senescence-associated heterochromatin foci (SAHF), are also observed in senescent cells. The condensed chromatin in senescent cells contributes to the stable repression of proliferation-promoting genes [4]. Increasing number of proteins have been reported to be involved in the chromatin changes during the senescence process [5]. However, little is known about how the epigenetic factors are involved in and contribute to the senescence pathway.

MCAF1 (also known as hAM or ATF7IP) is a transcriptional cofactor that was originally identified as a binding protein of the transcription factor ATF7 [6]. In addition, MCAF1 associates with general transcription factors [6], RNA polymerase II [6,7], and a transcriptional activator SP1 [8]. While MCAF1 associates with the transcriptional apparatus, it also interacts with a methyl-CpG binding protein MBD1 and a H3K9 methyltransferase SETDB1 to form heterochromatin [9,10], suggesting that MCAF1 may function as both a transcriptional activator and a repressor depending on the situation. Biochemical analysis revealed that MCAF1 is an enzymatic cofactor of SETDB1. SETDB1 itself has ability to mono- and di-methylates H3K9, but in the presence of MCAF1 it can also tri-methylate H3K9 [9]. In the cancer cell line C33a, MCAF1, MBD1, and SETDB1 co-localize at the H3K9me3-containing heterochromatin region [8,11]. MCAF1 contains the SUMO-interacting motif (SIM) which preferentially binds to SUMO2/3 [12]. Modification of MBD1 with SUMO2/3 is considered to be required for the recruitment of the MCAF1/SETDB1 complex to DNA-methylated loci to form heterochromatin [11]. Although MCAF1 is overexpressed in various types of cancers [7], the biological significance of MCAF1 remains largely unknown.

Here, we find that, in the human primary diploid fibroblasts IMR90, MCAF1 localizes to PML bodies, but not to H3K9me3-containing heterochromatin. We demonstrate that siRNA-mediated knockdown of MCAF1 in IMR90 cells induces premature senescence. MCAF1 knockdown activates the expression of the cdk inhibitors p16 and p21, dephosphorylates RB, and represses a subset of cell cycle genes. Moreover, core histones and the linker histone H1 are downregulated at both mRNA and protein levels in MCAF1-depleted cells. During senescence induction by activated Ras, the MCAF1 protein level is constant. However, MCAF1 further accumulates in PML bodies in senescent cells by binding to SUMO2/3 through the SIM, implying that sequestration of MCAF1 to PML bodies is necessary for the cells to enter the senescent state. Taken together, these data suggest that MCAF1 is an important regulator of cellular senescence whose activity may be regulated by SUMO.

## Material and Methods

### Cell culture

IMR90 cells were purchased from ATCC (catalog no. CCL-186) and cultured in DMEM supplemented with 10% FBS. For senescence induction, IMR90 ER: Ras cells [13] were treated with 100 nM 4-hydryoxytamoxifen (4-OHT) for 6 days.

### Plasmids, siRNAs, and transfection

The cDNA for wild type and D968A mutant of MCAF1 were inserted into the episomal vector pEBMulti (Wako) together with monomeric EGFP. Plasmid DNAs were transfected with Fugene HD (Roche) for 48 hr.

siRNAs used are Flexitube siRNA (SI04249455 for MCAF1-1, Qiagen), and SMART pool ON-TARGET plus (J-019289-05, J-019289-06, J-019289-07, J-019289-08 for MCAF1-2, Thermoscientific). The siRNA targeted to luciferase (GL3) was used as a control [7]. The siRNAs (2.5 nM) were transfected with the RNAiMAX transfection reagent (Life technologies) every 3 days.

### SA-β-gal assay and EdU incorporation assay

SA-β-gal assay was performed using the Senescence Detection Kit (BioVision). For EdU incorporation assay, Click-iT EdU Alexa Fluor 488 Imaging kit was used (Life technologies). EdU incorporation was carried out for 1 hr at 37^o^C at a final concentration of 10 µM EdU.

### Immunofluorescence

Cells were fixed with 4% paraformaldehyde in PBS for 10 min at room temperature and permeabilized with 0.2% triton X-100 in PBS for 5 min on ice. After blocking with 0.5% BSA in PBS for 30 min at R.T, the cells were sequentially incubated with a primary antibody followed by an appropriate secondary antibody. DNA was stained with DAPI and the cells were mounted under coverslips. The cells were analyzed using an Olympus IX71 microscope and the Lumina Vision software. Primary antibodies used are anti-MCAF1 [10], anti-PML (PG-M3, Santa cruz), anti-SUMO2/3 (3H2) [11], anti-macroH2A (39594, Active motif), anti-GFP (A11122, Life technologies), and anti-H3K9me3 (2F3) [14].

### RT-qPCR

Total RNA was extracted from cells with TRIzol reagent (Life technologies). RT reaction was carried out with the ReverTraAce qPCR RT kit (Toyobo). For analyzing histone gene expression, total RNA was treated with DNase I (Takara bio) before the RT reaction. qPCR analysis was performed using Thunderbird SYBR qPCR mix (Toyobo) and an ABI Prism 7500 system. Each experiment was carried out at least three times. The fold relative enrichment was quantified, together with normalization by the GAPDH level. Primer sequences used in this study are listed in Supplemental Table **S1**.

### Western blot analysis

Total cell lysate was prepared by directly suspending the cells in SDS sample buffer containing benzonase (Sigma). Antibodies used are anti-MCAF1 [10], anti-β-tubulin (T4026, Sigma), anti-p16 (JC8, Santa cruz), anti-p21 (C-19, Sancta cruz), anti-RB (554136, BD), anti-H1 (61202, Active motif), anti-H3 (ab1791, abcam), anti-H2A (39592, Active motif), anti-macroH2A (39594, Active motif), anti-HRas (F235, Santa cruz), and anti-GFP (A11122, Life technologies). Quantification of bands was done with the ImageQuant TL software (GE Healthcare). The band intensities were shown relative to the control, which was normalized to 1.

### MNase digestion assay

Extracted nuclei were suspended in MNase buffer (50 mM Tris-HCl pH 7.4, 320 mM sucrose, 4 mM MgCl_2,_ and 1 mM CaCl_2_) and incubated with MNase (Takara bio) for the indicated times. The reaction was stopped with 10 mM EGTA. Extracted DNA was analyzed by agarose gel electrophoresis.

### Microarray and gene set enrichment analysis (GSEA)

Gene expression microarray of total RNA from control or MCAF1-1 siRNA treated cells was performed by Dragon Genomics Center using an Affymetrix GeneChip Human Genome U133 Plus 2.0 array. GSEA was carried out as described [15].

### Statistical analyses

Data are presented as means±s.d. All statistical analyses were performed by a two-tailed Student’s *t*-test.

## Results

### MCAF1 localizes to PML bodies in human normal cell lines

**Figure 1:**
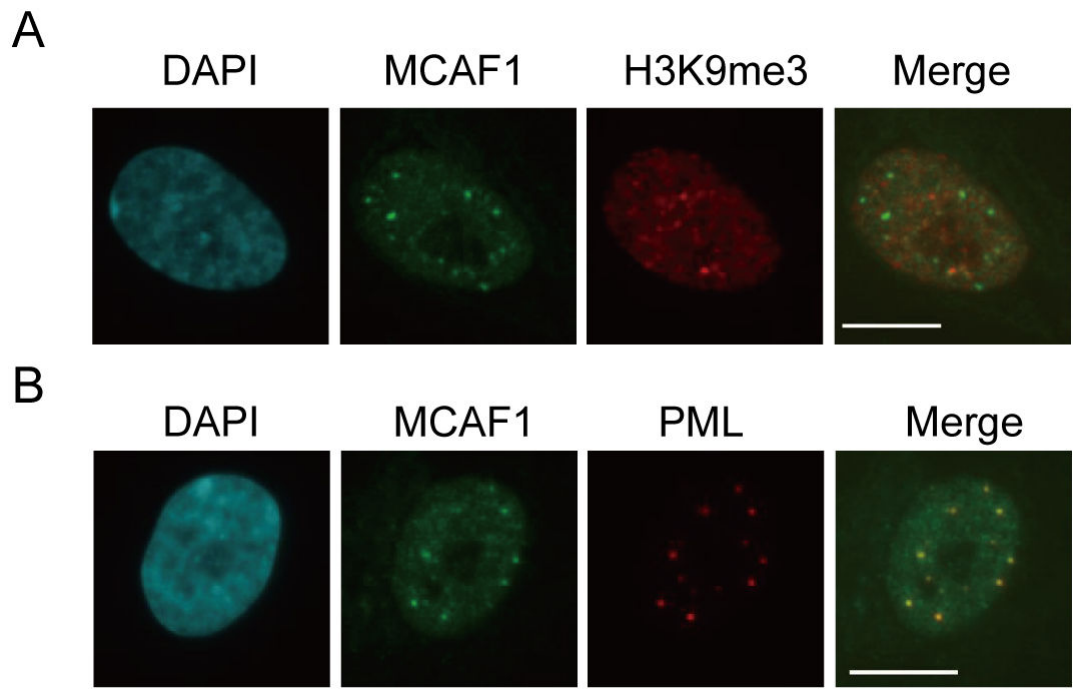
MCAF1 localizes to PML bodies in IMR90 fibroblasts. (**A**) Immunofluorescence analysis of endogenous MCAF1 and H3K9me3 in IMR90 cells. DNA was stained with DAPI. Scale bar: 10 µM. (**B**) Immunofluorescence of endogenous MCAF1 and PML in IMR90 cells. Scale bar: 10 µM.

To gain insight into the function of MCAF1, we analyzed subcellular localization of MCAF1 in various human cell lines. Immunofluorescence analysis with anti-MCAF1 antibody revealed that MCAF1 displayed two different localization patterns depending on the cell types; heterochromatin and discrete subnuclear foci. As was the case in cancerous C33a cells [11], MCAF1 colocalized with a heterochromatin marker H3K9me3 in HeLa and MCF7 cells (Figure **S1A**). In contrast, in human normal fibroblasts IMR90 and primary mammary epithelial cells HMEC, MCAF1 formed 10-20 discrete nuclear foci that do not accumulate H3K9me3 (Figure **1A**). Co-immunostaining analysis identified these foci as PML nuclear bodies (Figure **1B** and Figure **S1B**). MCAF1 and PML colocalization was not observed in HeLa cells (Figure **S1B**). PML bodies include various proteins involved in transcription, DNA repair, and cellular senescence, and SUMOylation is considered to be essential for the assembly of PML nuclear bodies [16]. MCAF1 does not seem to be a structural component of PML bodies, since MCAF1 knockdown did not affect PML body formation (Figure **S1C**).

### MCAF1 knockdown induces cell cycle arrest

**Figure 2:**
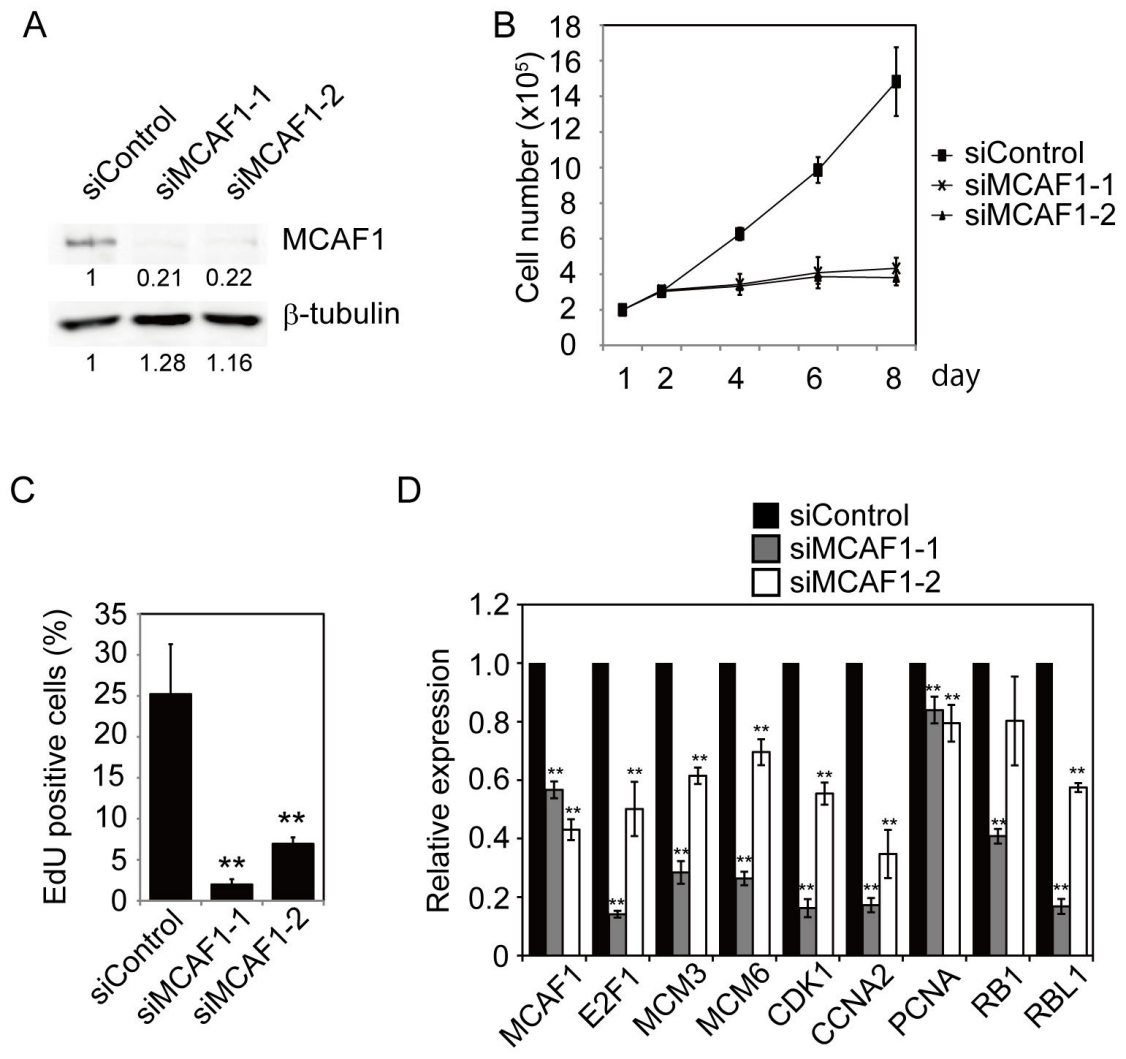
MCAF1 knockdown induces cell cycle arrest. (**A**) IMR90 cells were treated with the indicated siRNAs for 48 hr and analyzed by Western blotting with anti-MCAF1 and anti-β-tubulin antibodies. The images were quantitatively assessed by densitometry. (**B**) Growth curves for siControl, siMCAF1-1, and siMCAF1-2 cells. (**C**) EdU incorporation assay in control and MCAF1 knockdown cells. **P<0.01. (D) RT-qPCR analysis of the cell cycle genes at 48 hr after siRNA treatment. **P<0.01.

To examine the function of MCAF1 in PML bodies, we treated IMR90 cells with two independent siRNAs specific for MCAF1. Knockdown of MCAF1 protein was confirmed at 48 hr after siRNA treatment by Western blot analysis (Figure **2A**). MCAF1 knockdown severely impaired cell proliferation (Figure **2B**) and reduced incorporation of a thymidine analog EdU (Figure **2C**). As MCAF1 is a transcriptional regulator, we sought to identify target genes of MCAF1 by microarray analysis. IMR90 cells were treated with control or MCAF1 siRNAs for 48 hr, total RNA was extracted, and gene expression patterns were compared between control and MCAF1 knockdown cells by microarray and the gene set enrichment analysis. The results show that cell cycle genes were significantly downregulated in MCAF1 knockdown cells (Figure **S2**). To independently confirm the results, we performed RT-qPCR analysis. Again, the cell cycle genes, such as E2F1, MCMs, and CDK1, were significantly decreased by both siRNAs for MCAF1 (Figure **2D**), indicating that a subset of cell cycle genes are downstream targets of MCAF1 in IMR90 cells.

### Knockdown of MCAF1 induces premature senescence

**Figure 3:**
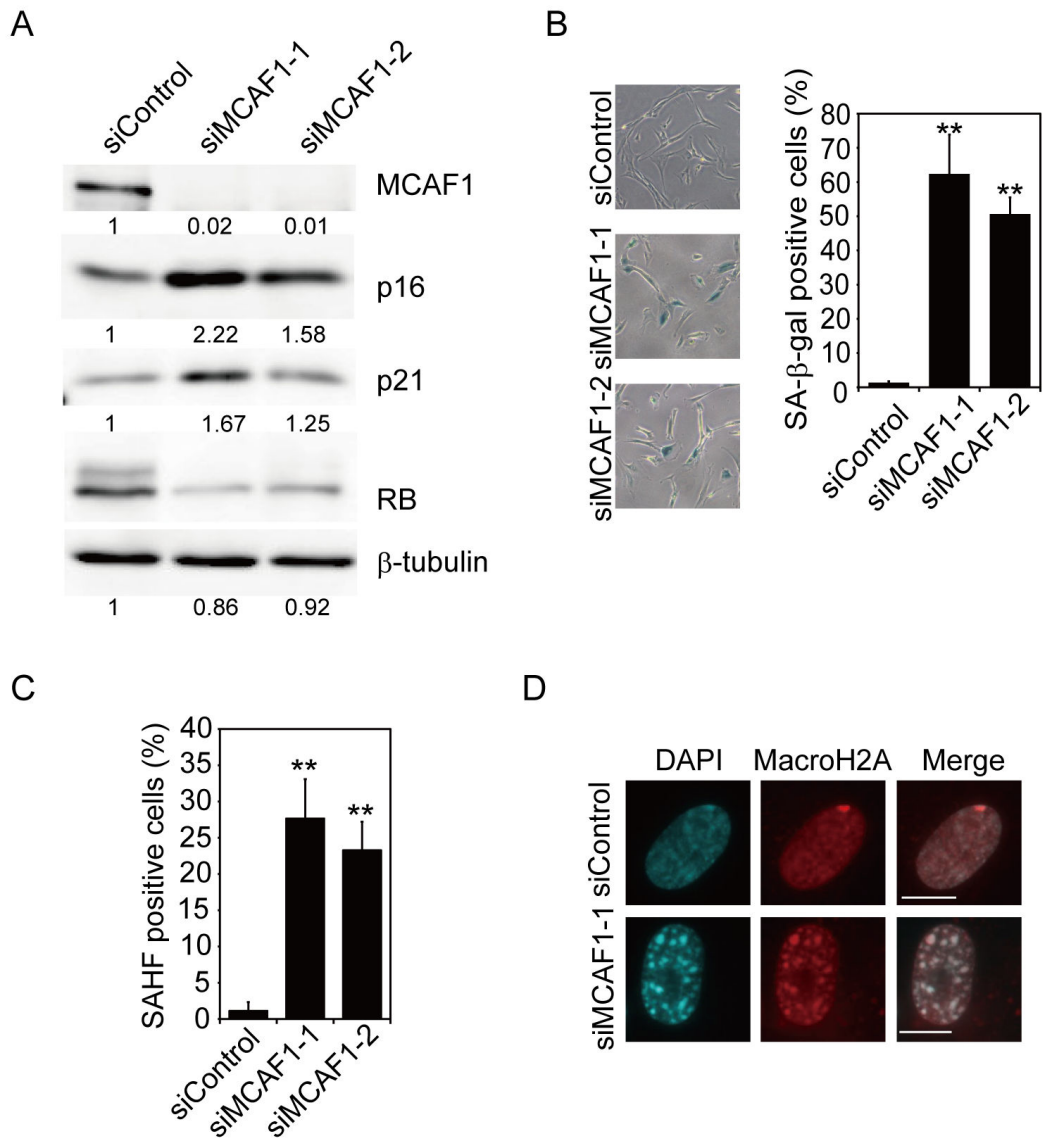
Knockdown of MCAF1 induces premature senescence. (**A**) Western blot analysis of the cdk inhibitors p16 and p21 and RB proteins in control and MCAF1 knockdown IMR90 cells. The images were quantitatively assessed by densitometry. (**B**, **C**) IMR90 cells were treated with the indicated siRNAs, and analyzed for SA-β-gal activity (B) or the formation of SAHF (C) at 8 days after siRNA treatment. **P<0.01. (**D**) Immunofluorescence analysis of MCAF1 and MacroH2A in control and SAHF-positive MCAF1 knockdown cells. DNA was stained with DAPI. Scale bar: 10 µM.

We also found that the cdk inhibitors p16 and p21 were upregulated at both mRNA and protein levels (Figure 3**A** and **S3**), and RB protein was dephosphorylated in MCAF1 knockdown cells (Figure **3A**). As cdk inhibitors play important roles in cellular senescence [17], we investigated if MCAF1 knockdown induced premature senescence. As shown in Figure **3B**, MCAF1 knockdown cells were positive for SA-β-gal activity. To further confirm that MCAF1 depletion induced senescence, we tested the formation of SAHF in MCAF1 knockdown cells. DAPI staining showed that approximately 30% of MCAF1 knockdown cells displayed nuclear foci that resemble SAHF (Figure **3C**). SAHF are facultative heterochromatin that is enriched for heterochromatin markers such as H3K9me3, HP1, and macroH2A [5]. The SAHF-like structure in MCAF1 knockdown cells were positive for macroH2A (Figure **3D**) and H3K9me3 (Figure **S4**), indicating that MCAF1 depletion induced SAHF formation. Collectively, these results indicate that MCAF1 knockdown activates the Rb pathway to trigger premature senescence.

### Reduction of histone gene expression in MCAF1 knockdown cells

**Figure 4:**
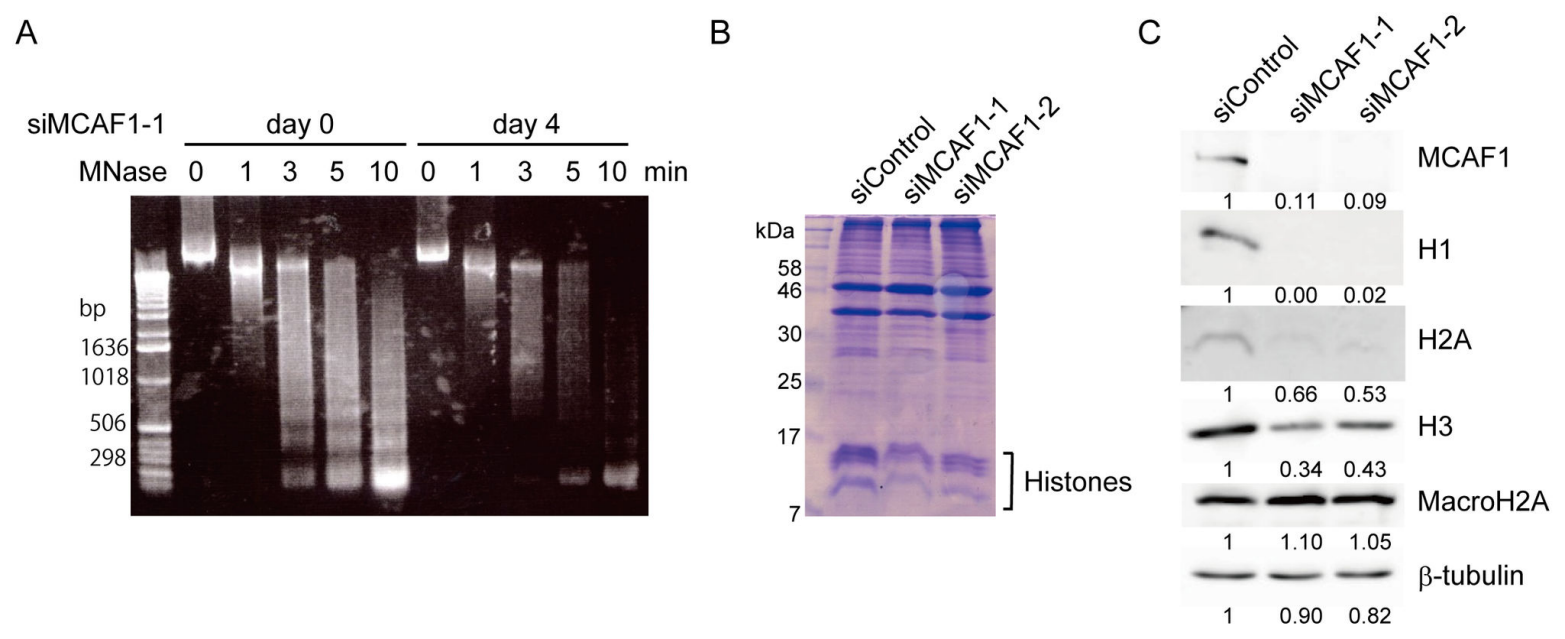
Histones are decreased at both mRNA and protein levels in MCAF1 knockdown cells. (**A**) Nuclei from control and MCAF1 knockdown IMR90 cells at 4 days after siRNA treatment were digested with MNase for the indicated times and analyzed by agarose gel electrophoresis. (**B**) Triton-insoluble fraction at 8 day after siRNA treatment was analyzed by SDS-page followed by CBB staining. (**C**) Western blot analysis of total cell lysates from the indicated cells at 8 days after siRNA treatment. The images were quantitatively assessed by densitometry.

We further investigated the mechanism by which MCAF1 knockdown induces premature senescence. As MCAF1 regulates chromatin state by associating with various proteins, we tested whether chromatin structure is affected in MCAF1 knockdown cells by micrococcal nuclease (MNase) digestion assay. Cell nuclei were collected from control and MCAF1 knockdown cells at 4 days after siRNA treatment. After the nuclei were treated with MNase, DNA was extracted and analyzed by agarose gel electrophoresis. As shown in Figure **4A**, in control cells, MNase digestion generated nucleosome ladders (Figure **4A**, left). Interestingly, in MCAF1 knockdown cells, MNase digestion produced fewer nucleosomes compared to control cells (Figure **4A**, right). As the reduction of undigested DNA in MCAF1 knockdown cells was comparable to that in control cells, these results suggest that the amount of nucleosomes is reduced in MCAF1 knockdown cells.

We then tested the levels of histone proteins in MCAF1 knockdown cells at 8 days after siRNA treatment. The CBB staining after SDS-PAGE showed that the levels of core histone proteins in MCAF1-depleted cells were decreased compared to control cells (Figure **4B**). Western blot analysis also showed that the amount of the core histones H2A and H3 and the linker histone H1 were significantly diminished in MCAF1 knockdown cells (Figure **4C**). However, the variant histone macroH2A was slightly increased in MCAF1 knockdown cells (Figure **4C**), which is consistent with the fact that macroH2A is upregulated in senescent cells [18]. To next investigate whether histone genes are downregulated at a transcriptional level, using RT-qPCR analysis, we analyzed the expression of total 9 histone genes from the histone gene cluster on chromosome 1 (Figure **S5A**). The expression of 8 out of 9 histone genes was downregulated in MCAF1-depleted cells at 48 hr after siRNA treatment. The expression of the variant histone genes macroH2A and H3.3A, which are not located on the histone gene cluster, were not decreased by MCAF1 knockdown (Figure **S5B**). These results suggest that histone genes in the histone gene cluster are simultaneously regulated by MCAF1.

### MCAF1 localizes to PML bodies through binding to SUMO2/3

**Figure 5:**
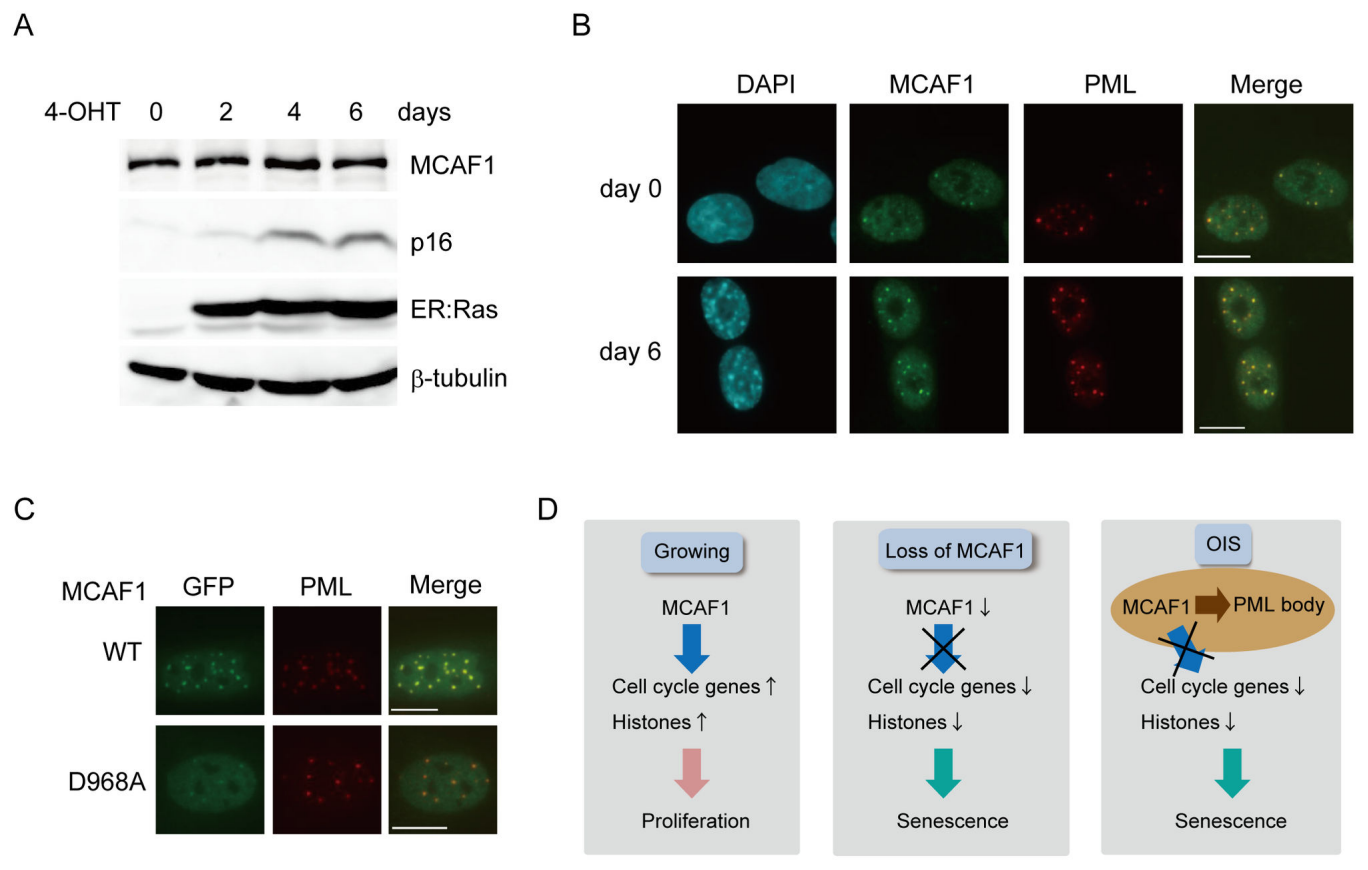
MCAF1 localizes to PML bodies through binding to SUMO2/3. (**A**) Western blot analysis of total cell lysates at the indicated times after ER: Ras induction. (**B**) Immunofluorescence analysis of MCAF1 and PML at 0 and 6 days after ER: Ras induction. Scale bar: 10 µM. (**C**) Immunofluorescence analysis of WT and D968 mutant of MCAF1 with anti-GFP antibody in IMR90 cells. Scale bar: 10 µM. (**D**) Role of MCAF1 in cellular senescence. In growing cells, MCAF1 maintains cell proliferation by activating transcription of cell cycle and histone genes (left). MCAF1 knockdown downregulates cell cycle and histone genes, and results in premature senescence (middle). In oncogene-induced senescent (OIS) cells, MCAF1 is recruited to PML bodies through binding to SUMO2/3. This may inhibit MCAF1 function to maintain expression of cell cycle genes and histones (right).

As MCAF1 depletion induces premature senescence, we then addressed whether MCAF1 protein level decreases during the senescence process. We induced senescence by expression of 4-OHT-inducible activated Ras (H-RasV12) [13]. Western blot analysis showed that the total amount of MCAF1 protein was constant until day 6 after 4-OHT treatment when cells became fully senescent (Figure **5A**). However, immunofluorescence analysis showed that MCAF1 was further accumulated in PML bodies in SAHF-positive senescent cells compared to in control cells (Figure **5B** and quantitatively shown in Figure **S6**). Similarly, MCAF1 accumulated to PML bodies in replicatively senescent cells (Figure **S7**), suggesting that accumulation of MCAF1 in PML body is a general feature of senescent cells.

We then investigated the mechanism by which MCAF1 is recruited to PML bodies. SUMO2/3 are also known to be accumulated in PML bodies in senescent cells (Figure **S8A**) [19]. As MCAF1 interacts with SUMO2/3 through the SIM [11], it was supposed that MCAF1 is recruited to PML bodies through binding to SUMO2/3. To address this possibility, we expressed EGFP-tagged wild type and the SIM mutant (D968A) [11] of MCAF1 in IMR90 cells and analyzed their localization by immunofluorescence analysis with anti-GFP antibody (Figure **S8B**). The wild type MCAF1 clearly colocalized with PML in transfected cells. In contrast, the SIM mutant of MCAF1 showed diffuse nuclear distribution, although very weak localization in PML bodies was observed (Figure **5C**). These results suggest that the SUMO2/3 binding through the SIM is required for the PML body localization of MCAF1 in senescent cells.

## Discussion

In this report, we demonstrated that MCAF1 is involved in the regulation of cellular senescence in human primary fibroblast cells. Our data suggest that two factors are major causative for the senescent phenotypes in MCAF1 knockdown cells; the upregulation of the cdk inhibitors and the downregulation of the histone proteins. The cdk inhibitors p16 and p21, which are upregulated in MCAF1 knockdown cells, play important roles in cellular senescence by dephosphorylating RB to repress expression of cell cycle genes [1]. Overexpression of p16 or p21 has been shown to be sufficient to induce senescent phenotypes in fibroblasts [17]. Therefore, upregulation of these cdk inhibitors may be a major factor that triggered senescence in MCAF1 knockdown cells. Further study would be necessary to elucidate which upstream factors activate p16 and p21 in MCAF1 knockdown cells.

Histone protein levels are also associated with the senescence pathway. Core histones and the liker histone H1 are known to be decreased in senescent human cells [18,20,21]. However, it remains unclear whether the histone reduction in MCAF1 knockdown cells is a cause or a result of senescence. It has been reported that histone reduction in yeast shortened lifespan [22]. In addition, depletion of the specific histone H1 variant in cancer cells results in cell cycle arrest [23]. Furthermore, knockdown of the histone variants H2AZ or CENPA induces premature senescence [24,25]. Therefore, these findings suggest that histone reduction in MCAF1 knockdown cells may also be causative for the senescence phenotypes.

MCAF1 is a transcriptional cofactor that associates with various proteins involved in transcriptional regulation. However, very few target genes of MCAF1 has been identified so far. We have reported that MCAF1 is required for cancer cell proliferation by activating the transcription of the telomerase genes, TERT and TERC [7]. ChIP-seq analysis in melanoma cells identified the HoxA gene cluster as direct MCAF1 targets [26]. The ChIP-seq data also shows the enrichment of MCAF1 at the histone gene loci and a subset of cell cycle genes, which we showed as possible downstream targets of MCAF1. Therefore, it would be likely that the cell cycle and histone genes are direct transcriptional targets of MCAF1 that are necessary to maintain cell proliferation.

MCAF1 colocalizes with SETDB1 in PML bodies (data not shown), but not with H3K9me3 in IMR90 cells (Figure **1**). Several recent reports demonstrate the PML body localization of SETDB1 in ES cells and in early embryos [27,28]. Our preliminary data suggest that SETDB1 knockdown induces premature senescence. Conversely, SETDB1 is an oncogene whose overexpression in zebra fish abrogates the BRAF(V600E)-induced senescence [26]. Therefore, SETDB1 protein level is also correlated with cell proliferation and senescence in a similar manner with MCAF1. It remains to be elucidated whether the function of the MCAF1/SETDB1 complex in PML bodies is separable from that in heterochromatin in cancer cells. A recent study indicated that the global pattern of H3K9me3 unchanges upon SAHF formation during oncogene-induced senescence, but 3D repositioning of H3K9me3 is involved [29]. In addition, MCAF1 is not required to maintain the H3K9me3 level within SAHF structure (Figure **S4**). Therefore, MCAF1 and SETDB1-mediated H3K9 methylation is not likely to play major roles during the senescence program.

PML protein plays important roles in the senescence pathway through the p53 signaling, and overexpression of PML induces premature senescence [30,31]. The histone chaperon HIRA accumulates in PML bodies in senescent cells similarly to MCAF1, whereas HP1 localizes to PML bodies at earlier stages of senescence before entering SAHF [32]. How dynamic relocalization of the proteins contributes to the senescence remains largely unknown. As both knockdown and PML body recruitment of MCAF1 are connected with the same senescent phenotypes, sequestration of MCAF1 to PML bodies may be essential to inhibit MCAF1 function to activate transcription of cell cycle and histone genes during the senescence pathway (Figure **5D**). SUMO proteins seem to play important roles in this process. Overexpression of SUMO or the SUMO E3 ligase PIA, Sy, or knockdown of the SUMO isopeptidase SENP1 induced senescent-like phenotypes [19,33,34]. Our data show that SUMO2/3 appear to be required for the accumulation of MCAF1 in PML bodies. In cancer cells, MCAF1 is recruited to heterochromatin via the SIM through binding to SUMOylated MBD1 [11]. In senescent cells, there would be some candidates including PML protein itself, which are SUMOylated and recruit MCAF1 to the bodies.

To summarize, our data show that MCAF1 regulates expression of cell cycle and histone genes to maintain cell proliferation, and its inhibition or sequestration to PML bodies are connected with cellular senescence. Given that MCAF1 is overexpressed in various cancers, manipulation of MCAF1 activity or its localization would be a useful tool for anti-cancer therapy.

## Supporting Information

Figure S1 MCAF1 localizes to PML bodies in normal cells, but not in cancer cells. (**A**) Immunofluorescence analysis of MCAF1 and a heterochromatin marker H3K9me3 in the cancer cell lines HeLa and MCF7. (**B**) Immunofluorescence of MCAF1 and PML in the normal mammary epithelial cell line HMEC. (**C**) IMR90 cells were treated with siRNA against MCAF1 for 48 hr and analyzed by immunofluorescence with MCAF1 and PML antibodies.
(PDF)

Figure S2 Identification of cell cycle genes as MCAF1 targets by microarray analysis. (**A**) Gene set enrichment analysis was performed to identify gene sets which were downregulated in MCAF1 knockdown cells compared to control cells. A list of top 20 gene sets is shown. Majority of the gene sets downregulated by MCAF1 knockdown are related to the cell cycle process. (**B**) Representative results of GSEA of downregulated genes in MCAF1 knockdown cells.
(PDF)

Figure S3 The cdk inhibitors p16 and p21 are upregulated in MCAF1 knockdown cells. RT-qPCR analysis of p16 and p21 in control and MCAF1 knockdown cells at 2 days after siRNA treatment.
(PDF)

Figure S4 SAHF in MCAF1 knockdown cells are enriched for H3K9me3. Immunofluorescence analysis of MCAF1 and H3K9me3 in control and SAHF-positive MCAF1 knockdown cells.
(PDF)

Figure S5 The core histone and H1 genes are downregulated in MCAF1 knockdown cells. (**A**) RT-qPCR was performed to analyze expression of histone genes in control and MCAF1 knockdown cells at 48 hr after siRNA treatment. (**B**) RT-qPCR analysis of the variant histone genes H3.3A and macroH2A at 48 hr after siRNA treatment.
(PDF)

Figure S6 MCAF1 accumulates in PML body in Ras-induced senescent cells. Line-scan histograms of MCAF1 (green), PML (red), and DAPI (blue) in control (left) and Ras-induced senescent (right) cells. Note that the signal intensity of MCAF1 within PML body in the Ras-induced senescent cells is higher than that in control cells.
(PDF)

Figure S7 MCAF1 is accumulated in PML bodies in replicatively senescent cells. Old IMR90 cells which display SAHF were immunostained with antibodies against MCAF1 and PML.
(PDF)

Figure S8 SUMO2/3 are accumulated in senescent cells. (A) Immunofluorescence of SUMO2/3 and PML at 0 and 6 days after ER: Ras induction. (**B**) Western blot analysis to confirm the expression of monomeric EGFP-tagged wild type and the D968A mutant of MCAF1 in IMR90 cells.
(PDF)

Table S1 A list of primers used in this study. (DOC)
