# Supplementary figures and images for: The Transcriptional Cofactor MCAF1/ATF7IP Is Involved in Histone Gene Expression and Cellular Senescence
Source: PLoS One. 2013 Jul 30;8(7):e68478. doi: 10.1371/journal.pone.0068478 (PMC3728336; doi:10.1371/journal.pone.0068478)

A

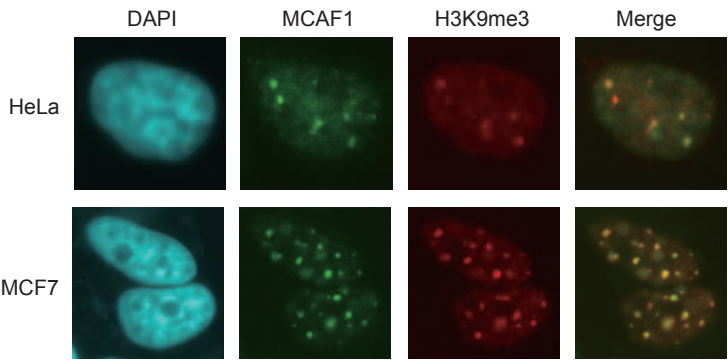

B

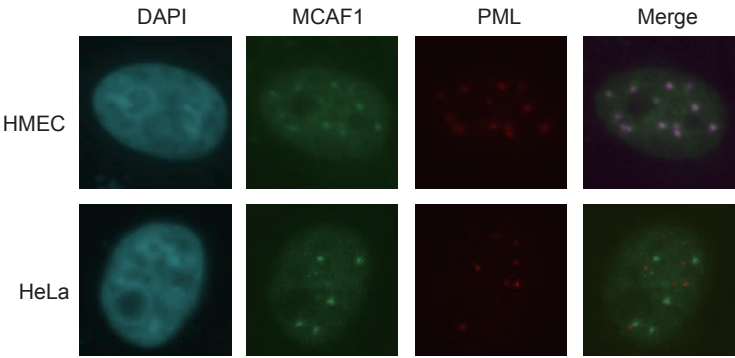

C

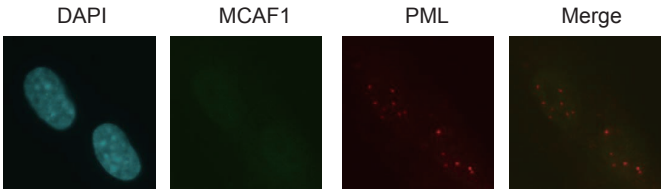

Supplement: Figure S1 — (A) Immunofluorescence analysis of MCAF1 and a heterochromatin marker H3K9me3 in the cancer cell lines HeLa and MCF7. (B) Immunofluorescence of MCAF1 and PML in the normal mammary epithelial cell line HMEC. (C) IMR90 cells were treated with siRNA against MCAF1 for 48 hr and analyzed by immunofluorescence with MCAF1 and PML antibodies. (PDF) [file pone.0068478.s001.pdf]

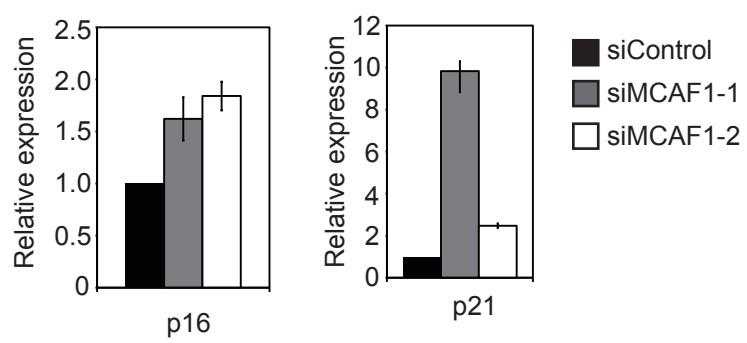

Supplement: Figure S3 — RT-qPCR analysis of p16 and p21 in control and MCAF1 knockdown cells at 2 days after siRNA treatment. (PDF) [file pone.0068478.s003.pdf]

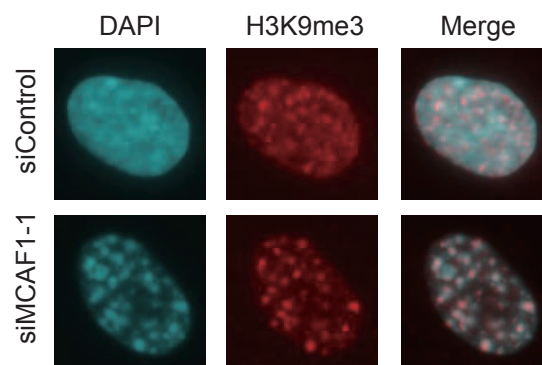

Supplement: Figure S4 — Immunofluorescence analysis of MCAF1 and H3K9me3 in control and SAHF-positive MCAF1 knockdown cells. (PDF) [file pone.0068478.s004.pdf]

A

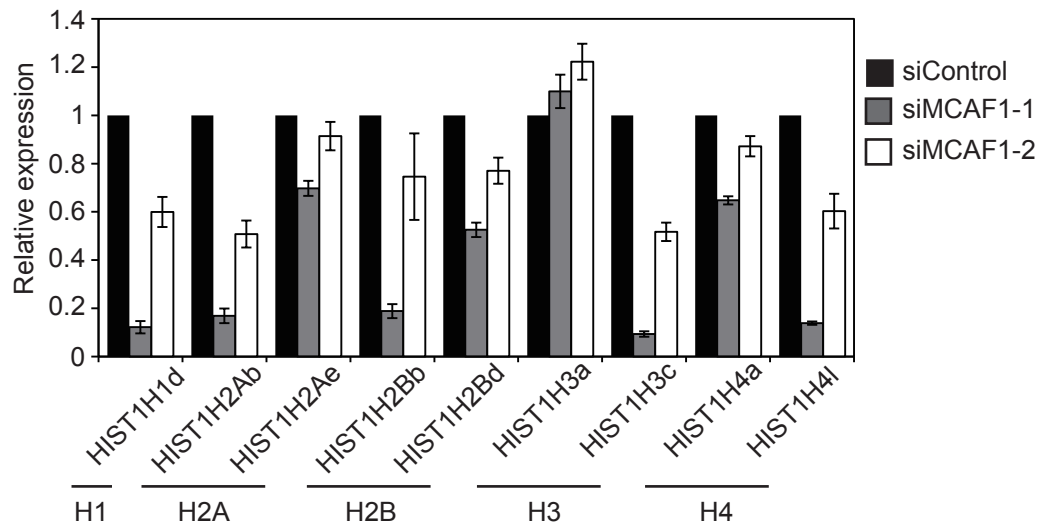

B

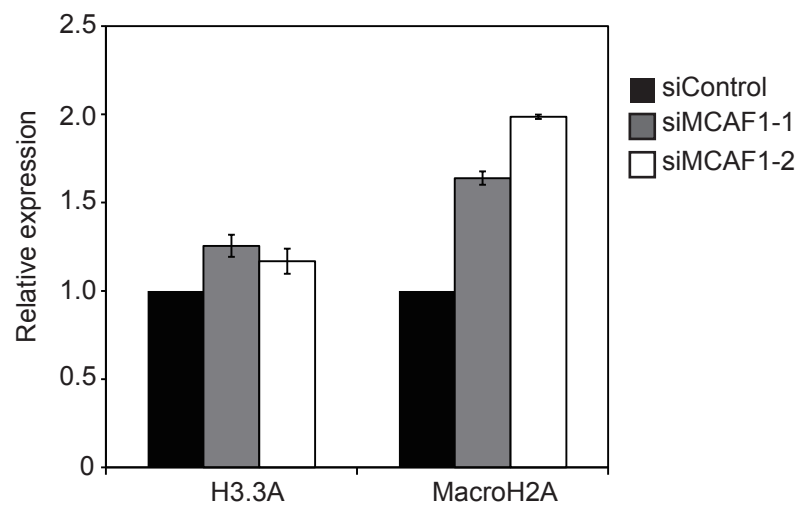

Supplement: Figure S5 — (A) RT-qPCR was performed to analyze expression of histone genes in control and MCAF1 knockdown cells at 48 hr after siRNA treatment. (B) RT-qPCR analysis of the variant histone genes H3.3A and macroH2A at 48 hr after siRNA treatment. (PDF) [file pone.0068478.s005.pdf]

day 0

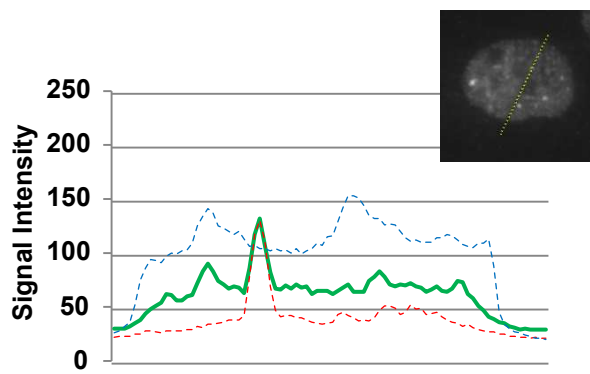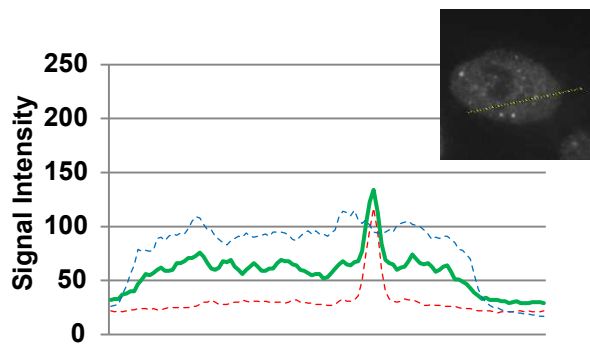

day 6

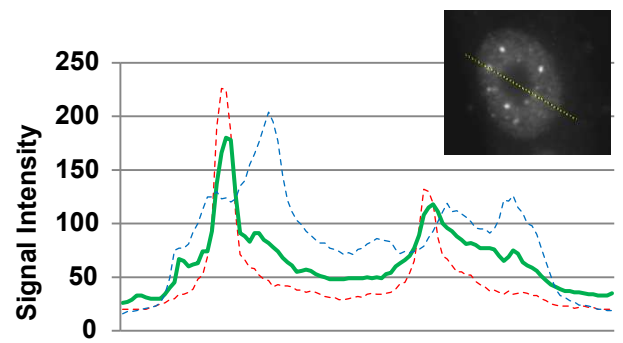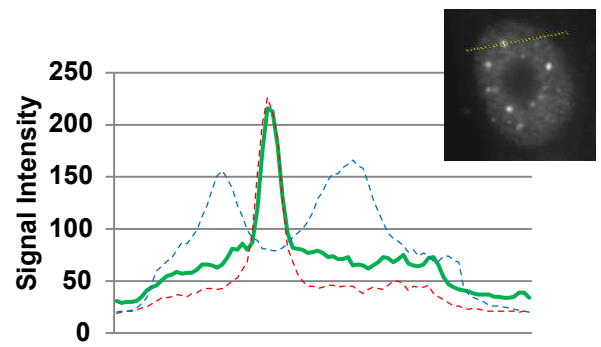

MCAF1  
PML  
DAPI

Supplement: Figure S6 — Line-scan histograms of MCAF1 (green), PML (red), and DAPI (blue) in control (left) and Ras-induced senescent (right) cells. Note that the signal intensity of MCAF1 within PML body in the Ras-induced senescent cells is higher than that in control cells. (PDF) [file pone.0068478.s006.pdf]

DAPI

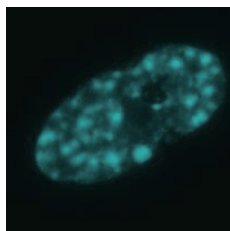

MCAF1

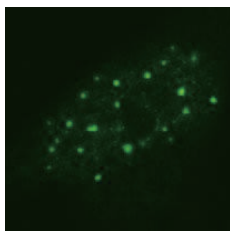

PML

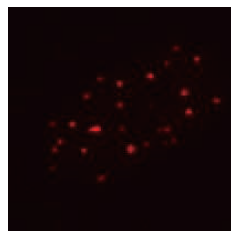

Merge

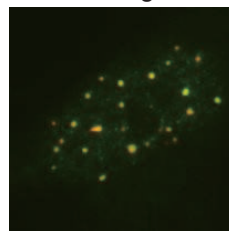

Supplement: Figure S7 — Old IMR90 cells which display SAHF were immunostained with antibodies against MCAF1 and PML. (PDF) [file pone.0068478.s007.pdf]

A

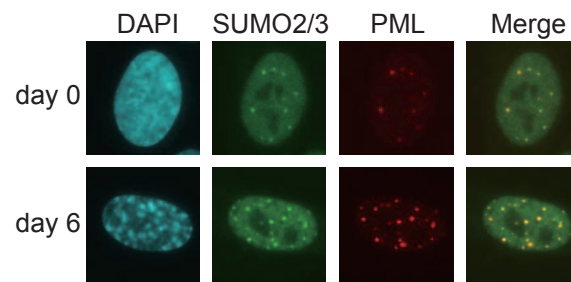

B

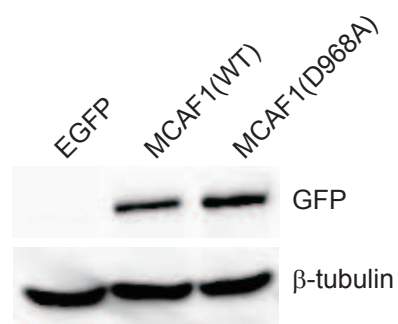

Supplement: Figure S8 — (A) Immunofluorescence of SUMO2/3 and PML at 0 and 6 days after ER: Ras induction. (B) Western blot analysis to confirm the expression of monomeric EGFP-tagged wild type and the D968A mutant of MCAF1 in IMR90 cells. (PDF) [file pone.0068478.s008.pdf]
