# Supplementary material for: The Transcriptional Cofactor MCAF1/ATF7IP Is Involved in Histone Gene Expression and Cellular Senescence
Source: PLoS One. 2013 Jul 30;8(7):e68478. doi: 10.1371/journal.pone.0068478 (PMC3728336; doi:10.1371/journal.pone.0068478)

A

| | GS DETAILS | ES | NES | NOM p-val | FDR q-val |
|---|---|---|---|---|---|
| 1 | MITOSIS | 0.65 | 2.22 | 0 | 0 |
| 2 | M_PHASE_OF_MITOTIC_CELL_CYCLE | 0.64 | 2.21 | 0 | 0 |
| 3 | REGULATION_OF_MITOSIS | 0.7 | 2.14 | 0 | 0 |
| 4 | SPINDLE | 0.69 | 2.1 | 0 | 0 |
| 5 | CELL_DIVISION | 0.79 | 2.1 | 0 | 0 |
| 6 | MITOTIC_CELL_CYCLE | 0.55 | 2.05 | 0 | 0 |
| 7 | CYTOKINESIS | 0.78 | 2.01 | 0 | 0.001 |
| 8 | CHROMOSOMEPERICENTRIC_REGION | 0.7 | 1.99 | 0 | 0.002 |
| 9 | CYTOKINE_BIOSYNTHETIC_PROCESS | 0.65 | 1.98 | 0 | 0.002 |
| 10 | CELL_CYCLE_PROCESS | 0.5 | 1.96 | 0 | 0.003 |
| 11 | CYTOKINE_METABOLIC_PROCESS | 0.65 | 1.96 | 0 | 0.002 |
| 12 | REGULATION_OF_CYTOKINE_BIOSYNTHETIC_PROCESS | 0.64 | 1.95 | 0 | 0.003 |
| 13 | MICROTUBULE_MOTOR_ACTIVITY | 0.77 | 1.92 | 0 | 0.004 |
| 14 | MICROTUBULE_CYTOSKELETON | 0.51 | 1.87 | 0 | 0.011 |
| 15 | CELL_CYCLE_CHECKPOINT_GO_0000075 | 0.6 | 1.87 | 0 | 0.011 |
| 16 | REPLICATION_FORK | 0.73 | 1.86 | 0 | 0.011 |
| 17 | M_PHASE | 0.52 | 1.84 | 0 | 0.013 |
| 18 | CELL_CYCLE_PHASE | 0.49 | 1.83 | 0 | 0.015 |
| 19 | NEGATIVE_REGULATION_OF_TRANSLATION | 0.71 | 1.82 | 0 | 0.017 |
| 20 | MITOTIC_CELL_CYCLE_CHECKPOINT | 0.69 | 1.81 | 0 | 0.018 |

B

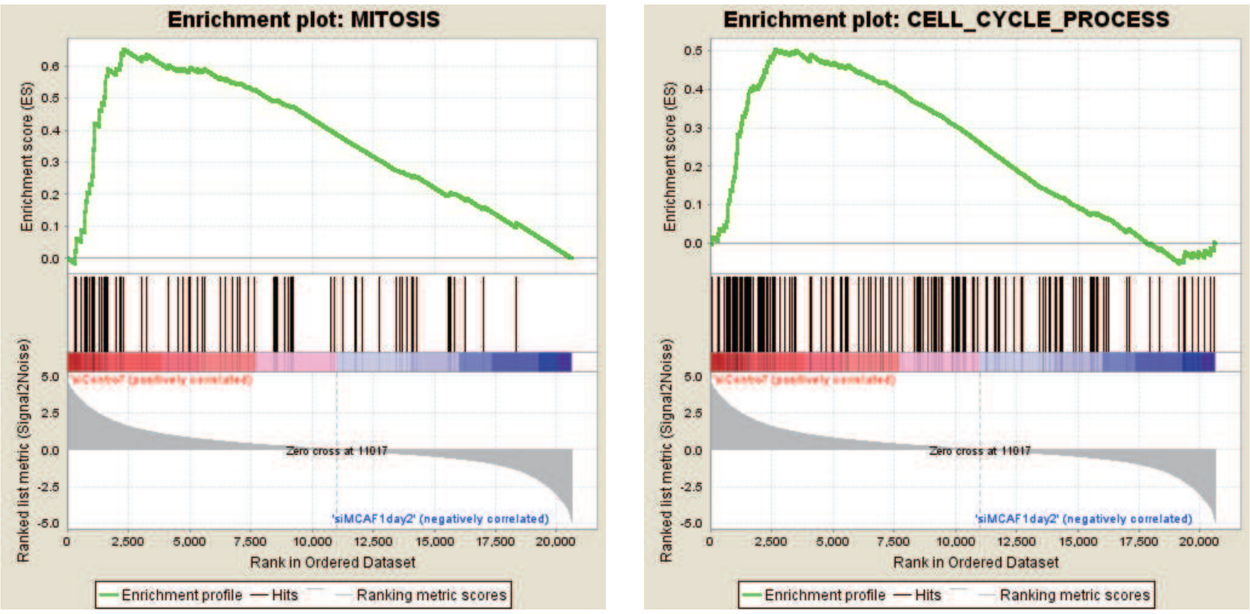

Supplement: Figure S2 — (A) Gene set enrichment analysis was performed to identify gene sets which were downregulated in MCAF1 knockdown cells compared to control cells. A list of top 20 gene sets is shown. Majority of the gene sets downregulated by MCAF1 knockdown are related to the cell cycle process. (B) Representative results of GSEA of downregulated genes in MCAF1 knockdown cells. (PDF) [file pone.0068478.s002.pdf]
