# Supplementary material for: The Transcriptional Cofactor MCAF1/ATF7IP Is Involved in Histone Gene Expression and Cellular Senescence
Source: PLoS One. 2013 Jul 30;8(7):e68478. doi: 10.1371/journal.pone.0068478 (PMC3728336; doi:10.1371/journal.pone.0068478)
Supplement: Table S1 — (DOC) [file pone.0068478.s009.doc]

**Table S1. A list of primers used in this study.**

| Primer | Sequence |
| --- | --- |
| MCAF1-F | 5´-AGTTATATCGCAAAATGAAACGTG-3´ |
| MCAF1-R | 5´-TCCTCAGGTTTGTTGTCCTTTT-3´ |
| E2F1-F | 5’-GCCATCCAGGAAAAGGTG-3’ |
| E2F1-R | 5’-GTCAGCCGAGTGGCTCAG-3’ |
| MCM3-F | 5’-GGGTGGAACGAGACCTAGAA-3’ |
| MCM3-R | 5’-AGACTTGGCAACGGATGG-3’ |
| MCM6-F | 5’-CAGCTAAGAGCCAATTTCTCAAG-3’ |
| MCM6-R | 5’-GGACGCTTTACCACTGGTGT-3’ |
| CDK1-F | 5’-TGGATCTGAAGAAATACTTGGATTCTA-3’ |
| CDK1-R | 5’-CAATCCCCTGTAGGATTTGG-3’ |
| CCNA2-F | 5’-GGTACTGAAGTCCGGGAACC-3’ |
| CCNA2-R | 5’-GAAGATCCTTAAGGGGTGCAA-3’ |
| PCNA-F | 5’-TGGAGAACTTGGAAATGGAAA-3’ |
| PCNA-R | 5’-GAACTGGTTCATTCATCTCTATGG-3’ |
| RB1-F | 5’-GGATCAGATGAAGCAGATGGA-3’ |
| RB1-R | 5’-GCATTCGTGTTCGAGTAGAAGTC-3’ |
| RBL1-F | 5’-TTGGCGAATCAGGACCATA-3’ |
| RBL1-R | 5’-GGTGAGCCTGGCTGTTGT-3’ |
| P16-F | 5’-CAACGCACCGAATAGTTACG-3’ |
| P16-R | 5’-CTGCCCATCATCATGACCT-3’ |
| P21-F | 5’-CGAAGTCAGTTCCTTGTGGAG-3’ |
| P21-R | 5’-ACCTTGGCAGCAACTGGAT-3’ |
| HIST1H1d-F | 5’-CTGCTCCACTTGCTCCTACC-3’ |
| HIST1H1d-R | 5’-GCCTTCTTCGCCTTTTTCTT-3’ |
| HIST1H2Ab-F | 5’-ATAAACTCTTGGGGCGTGTG-3’ |
| HIST1H2Ab-R | 5’-TTTCCCTTGGCCTTATGATG-3’ |
| HIST1H2Ae-F | 5’-CCAAGGGCAAGTGAAATGAT-3’ |
| HIST1H2Ae-R | 5’-AGCCTTTGGTTTCTGGGACT-3’ |
| HIST1H2Bb-F | 5’-TAAGTCTGCTCCAGCCCCTA-3’ |
| HIST1H2Bb-R | 5’-CTGCGCTTACGCTTCTTACC-3’ |
| HIST1H2Bd-F | 5’-TCAGAAGAAGGACGGGAAGA-3’ |
| HIST1H2Bd-R | 5’-GGAATTCATGATCCCCATTG-3’ |
| HIST1H3a-F | 5’-GAGGGCGTGATTACTGTGGT-3’ |
| HIST1H3a-R | 5’-AGGTGGTGGCTCTGAAAAGA-3’ |
| HIST1H3c-F | 5’-CTACCTGGTGGGACTCTTCG-3’ |
| HIST1H3c-R | 5’-CGTTTAGCGTGAATAGCGCA-3’ |
| HIST1H4a-F | 5’-CTTTATGGCTTTGGCGGTTA-3’ |
| HIST1H4a-R | 5’-TTCAGAAATGCAAGCTGTGG-3’ |
| HIST1H4l-F | 5’-CAAAGTTCTGCGCGACAACA-3’ |
| HIST1H4l-R | 5’-TAAGAACTCCGCGTGTCTCC-3’ |
| H3.3A-F | 5’-GCCATCTTTCAATTGTGTTCG-3’ |
| H3.3A-R | 5’-GCCATGGTACAGAGACCTCCT-3’ |
| macroH2A-F | 5’-CAGTCCTCTCCACCAAGAGC-3’ |
| macroH2A-R | 5’-TCTTCTCCAGCGTGTTTCCT-3’ |
| GAPDH-F | 5´-ACACCCACTCCTCCACCTTT-3´ |
| GAPDH-R | 5´-TAGCCAAATTCGTTGTCATACC-3´ |
